# Contribution of Heritability and Epigenetic Factors to Skeletal Muscle Mass Variation in United Kingdom Twins

**DOI:** 10.1210/jc.2016-1219

**Published:** 2016-05-04

**Authors:** Gregory Livshits, Fei Gao, Ida Malkin, Maria Needhamsen, Yudong Xia, Wei Yuan, Christopher G. Bell, Kirsten Ward, Yuan Liu, Jun Wang, Jordana T. Bell, Tim D. Spector

**Affiliations:** Department of Twin Research and Genetic Epidemiology (G.L., M.N., W.Y., C.G.B., K.W., J.T.B., T.D.S.), King's College London, London SE1 7EH, United Kingdom; Human Population Biology Research Unit (G.L., I.M.), Department of Anatomy and Anthropology, Sackler Faculty of Medicine, Tel Aviv University, Tel Aviv 6997801, Israel; Beijing Genomics Institute-Shenzhen (F.G., Y.X., Y.L., J.W.), Shenzhen 518083, China; King Abdulaziz University (J.W.), Jeddah 22254, Saudi Arabia; and Department of Biology (J.W.) and The Novo Nordisk Foundation Center for Basic Metabolic Research (J.W.), University of Copenhagen, Copenhagen DK-2200, Denmark

## Abstract

**Context:**

Skeletal muscle mass (SMM) is one of the major components of human body composition, with deviations from normal values often leading to sarcopenia.

**Objective:**

Our major aim was to conduct a genome-wide DNA methylation study in an attempt to identify potential genomic regions associated with SMM.

**Design:**

This was a mixed cross-sectional and longitudinal study.

**Setting:**

Community-based study.

**Participants:**

A total of 1550 middle-aged United Kingdom twins (monozygotic [MZ] and dizygotic [DZ]), 297 of which were repeatedly measured participated in the study.

**Main Outcome Measure:**

Appendicular lean mass assessed using dual-energy X-ray absorptiometry technology, and methylated DNA immunoprecipitation sequencing DNA methylation profiling genome-wide were obtained from each individual.

**Results:**

Heritability estimate of SMM, with simultaneous adjustment for covariates obtained using variance decomposition analysis, was h^2^ = 0.809 ± 0.050. After quality control and analysis of longitudinal stability, the DNA methylation data comprised of 723 029 genomic sites, with positive correlations between repeated measurements (R_repeated_ = 0.114–0.905). Correlations between MZ and DZ twins were 0.51 and 0.38 at a genome-wide average, respectively, and clearly increased with R_repeated_. Testing for DNA methylation association with SMM in 50 discordant MZ twins revealed 36 081 nominally significant results, of which the top-ranked 134 signals (*P* < .01 and R_repeated_ > 0.40) were subjected to replication in the sample of 1196 individuals. Seven SMM methylation association signals replicated at a false discovery rate less than 0.1, and these were located in or near genes *DNAH12*, *CAND1*, *CYP4F29P*, and *ZFP64*, which have previously been highlighted in muscle-related studies. Adjusting for age, smoking, and blood cell heterogeneity did not alter significance of these associations.

**Conclusion:**

This epigenome-wide study, testing longitudinally stable methylation sites, discovered and replicated a number of associations between DNA methylation at CpG loci and SMM. Four replicated signals were related to genes with potential muscle functions, suggesting that the methylome of whole blood may be informative of SMM variation.

## Abstract (precis)

“We conducted a genome-wide DNA methylation study in an attempt to identify potential genomic regions associated with skeletal muscle mass variation. Four replicated signals were found close to genes with potential muscle functions.”

Lean body mass (LBM), in particular skeletal muscle mass (SMM) is one of the 3 major components of body composition, which also includes fat body mass and bone mass. As for the other 2 components, SMM is highly important for normal physiology and metabolism, and deviations from normal values are often associated with various pathological conditions, notably sarcopenia, particularly in women (1). Sarcopenia is as a rule defined as age-related reduction in muscle mass and muscle strength, and affects women more than men with the prevalence as high as 30% for those above 60 years old (2, 3). In the elderly, the loss of LBM is correlated with profound physical impairment and disability with severe clinical consequences, including mobility loss, osteoporosis, increased fracture risk, dyslipidemia, insulin resistance, and increased mortality (4).

Despite its clinical significance and LBM has a strong familial component (5–8), replicated and confirmed specific genetic polymorphisms are rare and can explain only a minor part of the muscular mass variation (9–12). To date, no confirmed genotypes are associated with accelerated sarcopenia or frailty, or can explain a significant part of the interindividual variation in SMM.

Twin studies have made a remarkable contribution to the understanding of the molecular basis of human complex traits via modern high-throughput genetic and genomic analyses, and recently epigenetic approaches (13, 14). There have been major advances in epigenetics related to carcinogenesis (15, 16), psychiatric diseases (17), rheumatoid arthritis (18, 19), and others. However, we are not aware of studies exploring the epigenetics of muscular mass variation, despite suggestions of its importance (20), and recent work has explored age-related differential methylation signatures in human skeletal muscle (21).

The main aim of this study was therefore to conduct a genome-wide DNA methylation study of a large sample of United Kingdom twins, using methylated DNA immunoprecipitation sequencing (MeDIP-seq) in an attempt to identify potential genomic regions where DNA methylation levels are associated with SMM variation, with a secondary aim to obtain good estimates of heritability.

## Materials and Methods

### Study sample

The data examined in the present study were from the TwinsUK Adult Twin Registry, described in detail elsewhere (22). The sample had been collected from the general population through national media campaigns in the United Kingdom without ascertainment for any of individual characteristics, diseases or traits. In the present study, 1196 individuals (119 dizygotic [DZ] and 428 monozygotic [MZ] twin pairs and 102 singletons, 56 MZ and 46 DZ twins without sibling measurements) were included having all measurements of interest. All studied individuals were females with age range between 17 and 82 years and average 51.8 ± 13.7 years. The mean body mass index was 25.2 ± 4.7 kg/m^2^, and LBM ranged between 26.0 and 60.9 kg, with mean LBM = 39.9 ± 5.4 kg. All participants gave written informed consent before entering the study and the St Thomas' Hospital research ethics committee had approved the project.

### Muscle mass phenotype

All 3 major body composition components, ie, bone mineral density, fat body mass, and LBM, were measured by using standard whole body dual-energy X-ray absorptiometry method (5), following manufacturer's recommendations (QDR 4500W system; Hologic, Inc). Briefly, at installation, the manufacturer's engineer calibrated the instrument, and then daily quality control (QC) scans were performed using the spine phantom. Intrascanner reproducibility, expressed as a coefficient of variation from duplicate measurements in healthy volunteers 1 week apart, was 0.8% at the lumbar spine. Both twins within a pair were scanned on the same day. For the purposes of this study, we defined SMM as the sum of LBM measurements at the 4 limbs (appendicular lean mass), and not total LBM, which is biased by measurement of nonmuscular soft tissue, in particular viscera.

### Smoking scores

The present sample included 1100 individuals for whom the information on smoking habits was available. Of these, in 446 individuals were smokers, which included 320 current smokers and 126 exsmokers.

For blood cell composition, whole-blood cell (WBC) subtype counts were obtained for 441 individuals from the replication sample (n = 1196) using fluorescence-activated cell sorting (FACS) of peripheral blood (23). WBC subtype cell counts were available for 4 cell types: neutrophils, eosinophils, monocytes, and lymphocytes.

### DNA extraction and MeDIP-seq

Whole-blood samples (6 mL) were collected and stored at −80°C in EDTA tubes. Genomic DNA was extracted using the BACC3 Genomic DNA Extraction kit (Nucleon) and stored in TE buffer at −20°C. A total of 1.5 μg of genomic DNA was fragmented to a smear of 200–500 bp with the Bioruptor NGS System (Diagenode) and was subsequently end repaired, adenylated, and adapter ligated using the Paired-End DNA Sample Prep kit (Illumina). Methylated DNA was immunoprecipitation using the Magnetic Methylated DNA Immunoprecipitation kit (Diagenode) as previously described (24). After efficiency and sensitivity assessment by quantitative PCR, MeDIP-seq libraries were prepared by amplification (Platinum pfx DNA Polymerase kit; Invitrogen), purification (Agencourt Ampure Beads; Beckman Coulter) and validation (Agilent BioAnalyzer analysis) followed by high-throughput sequencing (Illumina HiSeq2000) that generated approximately 50 million, 50-bp single-end reads.

### MeDIP-seq DNA methylation quantification

After adapter and base quality trimming sequencing, reads were mapped to hg19 using BWA v0.5.9 (25). Alignments with low quality scores (Q < 10) and duplicates were filtered, which resulted in an average of 15 684 723 uniquely mapped reads that were subsequently extended to 350 bp to represent the average MeDIP fragment size. Fragments per kilobase per million ([number of mapped, extended reads per bin]/[million uniquely mapped reads per sample]) were quantified in bins of 500-bp (250-bp overlap) genome-wide using MEDIPS v1.6 (26).

### Design of the study and statistical analysis

The analysis of the data included the following stages and corresponding methods and was based on 3 nonoverlapping subsamples, selected from the total available sample.

The first sample was used for identification of longitudinally stable DNA methylation regions for downstream association analysis. For the selection of these methylation regions, we computed Pearson correlations between the all-available longitudinal measurements of the methylation within the individual. The bins displaying significant intraindividual correlations were considered as longitudinally stable, and from now on will be called longitudinally stable bin (lsBIN). We assumed that the lsBINs would also show significant correlation between twins, which could be regarded as an additional confirmation of their nonsporadic nature. To this end, 292 individuals having sequential measurements of methylation with the same processing batch were selected from the entire available sample (385 individuals having at least 2 methylation measurements each). Five of them had 2 pairs of appropriate sequential measurements each (4 individuals had 4 measurements and one 3 measurements with the same batch), the rest 2 measurements only. The measurements were taken with at least 3 years' time interval (mean, 7.0; SD, 1.2; range, 3.4–10.8). For the intrapair correlation, 104 pairs of MZ twins (219 pair of measurements) and 63 pairs of DZ twins (129 pair of measurements) with methylation measured at the same date and within the same batch were selected from the same total sample.

The second sample was used for initial identification of the bins potentially associated with SMM variation. For this aim, we selected 50 of the most discordant SMM pairs of MZ twins, implementing a simple formula dSMM_i_ = (dSMM_i1_ − dSMM_i1_)/0.5(dSMM_i1_ + dSMM_i1_). The 50 dSMM_i_ were explored for the corresponding methylation differences, dMTL_i_. Only lsBINs were used in this analysis. The DXA scans and methylation were taken with less than or equal to 12 months between both types of assessment.

The third sample was used for validation in an extended dataset. At this stage, lsBINs identified above were examined in the remaining sample including 1196 individuals in total (see above). We tested correlation between SMM and methylation level and used multiple regression analysis where SMM was considered a dependent variable, methylation level, age, and smoking as covariates. In a subset of this sample (n = 441) with available WBC, we also considered WBC subtype proportions for association with DNA methylation levels at the 7 top-ranked SMM-associated methylation signals and found no differences in the pattern of their association.

To evaluate the contribution of the additive genetic factors (heritability estimates, h^2^) to variation and covariation of SMM and methylation levels of the significantly associated bins, we carried out uni- and bivariate variance decomposition analysis, based on a classical polygenic concept of the quantitative trait inheritance. The analysis was conducted by MAN statistical package for family-based samples (http://www.tau.ac.il/∼idak/hid_MAN.htm).

### Functional genomic and CpG island (CGI) annotations

Annotations were obtained from University of California Santa Cruz (UCSC) (http://hgdownload.soe.ucsc.edu/goldenPath/hg19/database/cpgIslandExt.txt.gz). CGI shores were subsequently defined as 2-kb regions up- and downstream of CGI boundaries. Promoter (active, weak, and poised) and enhancer (strong and weak) chromatin states predicted by ChromHMM (27) for GM12878 (a lymphoblastoid cell line derived from blood) (28) were also obtained from UCSC (http://hgdownload.cse.ucsc.edu/goldenPath/hg19/encodeDCC/wgEncodeBroadHmm/wgEncodeBroadHmmGm12878HMM.bed.gz). Distribution of the methylation signals by the levels of their longitudinal stability, and extent of twin correlations among CGIs, promoters, and enhancers was compared using standard ANOVA and *t* tests.

## Results

### Heritability of SMM

The crude SMM measurements showed modest but statistically significant inverse correlation with age (r = −0.097, *P* = .006). The intraclass correlations of MZ and DZ twins for the age-adjusted SMM were high and significant: R_MZ_ = 0.799, *P* = .0001 and R_DZ_ = 0.366, *P* = .0008, respectively, suggestive of strong genetic influence. Indeed the heritability estimate obtained using variance decomposition analysis yielded h^2^ = 0.809 ± 0.050.

### Identification of longitudinally stable methylation signals

First, from the total 11 524 145 bins quantified genome-wide, those displaying zero methylation levels in more than 20% of the individuals were excluded, leaving 6 501 931 bins (56.4%) for further analysis (Table 1). Of these, only a minor portion of 723 029 bins (6.3% of the initial 11 524 145 bines) showed significant positive correlation between the longitudinal MeDIP-seq measurements within individuals, ranging between 0.114 and 0.905, with nominal *P* < .05.

**Table 1.** Summary Results of Testing for Longitudinal Stability of the Bins Methylation in 292 Individuals With 2 or More Repeated Measurements, Taken ≥4 Years Apart

Chr	N_ini_	N_filtered_	N_sign_	Max {R_repeated_}	Min {R_repeated_}	N_posit_	Corel.1	Corel.2	Corel.3	Max {R_MZ_}	Max {R_DZ_}	N_filtered_/N_ini_	N_sign_/N_ini_	N_posit_/N_ini_
1	997 003	556 000	64 467	0.823	−0.257	58 732	0.501	0.376	0.404	0.889	0.639	0.558	0.065	0.059
2	972 798	563 291	63 797	0.718	−0.256	57 973	0.518	0.365	0.416	0.831	0.690	0.579	0.066	0.060
3	792 090	447 367	48 947	0.766	−0.247	44 425	0.568	0.391	0.431	0.773	0.659	0.565	0.062	0.056
4	764 618	394 755	44 373	0.846	−0.218	40 143	0.582	0.395	0.430	0.934	0.625	0.516	0.058	0.053
5	723 662	394 564	44 526	0.753	−0.236	40 427	0.547	0.393	0.424	0.817	0.719	0.545	0.062	0.056
6	684 461	378 575	42 859	0.774	−0.261	38 963	0.555	0.420	0.447	0.841	0.620	0.553	0.063	0.057
7	636 555	374 256	46 089	0.776	−0.255	42 524	0.513	0.384	0.401	0.821	0.607	0.588	0.072	0.067
8	585 457	336 399	40 097	0.832	−0.255	36 713	0.569	0.390	0.432	0.888	0.623	0.575	0.068	0.063
9	564 854	274 602	34 249	0.764	−0.229	31 573	0.480	0.356	0.380	0.790	0.575	0.486	0.061	0.056
10	542 139	333 790	40 766	0.905	−0.229	37 579	0.505	0.370	0.401	0.934	0.568	0.616	0.075	0.069
11	540 027	312 132	37 634	0.826	−0.253	34 581	0.479	0.347	0.387	0.853	0.572	0.578	0.070	0.064
12	535 408	323 464	37 031	0.780	−0.239	33 766	0.499	0.365	0.390	0.872	0.746	0.604	0.069	0.063
13	460 680	216 253	24 730	0.743	−0.240	22 584	0.534	0.369	0.417	0.787	0.592	0.469	0.054	0.049
14	429 399	214 025	25 936	0.745	−0.230	23 792	0.531	0.399	0.412	0.836	0.599	0.498	0.060	0.055
15	410 126	210 537	26 333	0.802	−0.218	24 261	0.583	0.392	0.430	0.790	0.668	0.513	0.064	0.059
16	361 420	222 438	32 621	0.734	−0.244	30 675	0.457	0.365	0.368	0.789	0.565	0.615	0.090	0.085
17	324 781	229 520	31 730	0.755	−0.241	29 623	0.438	0.372	0.360	0.881	0.592	0.707	0.098	0.091
18	312 309	179 787	20 942	0.830	−0.223	19 148	0.525	0.394	0.426	0.892	0.500	0.576	0.067	0.061
19	236 516	179 218	28 430	0.801	−0.229	27 185	0.406	0.361	0.328	0.837	0.588	0.758	0.120	0.115
20	252 103	166 570	21 391	0.695	−0.220	19 873	0.435	0.329	0.364	0.748	0.534	0.661	0.085	0.079
21	192 520	88 201	12 546	0.738	−0.232	11 730	0.461	0.361	0.382	0.846	0.564	0.458	0.065	0.061
22	205 219	106 187	17 571	0.782	−0.243	16 759	0.413	0.376	0.319	0.797	0.538	0.517	0.086	0.082
ALL	11 524 145	6 501 931	787 065	0.905	−0.261	723 029	0.512	0.377	0.403	0.934	0.746	0.564	0.068	0.063

N_ini_, initial number of bins for chromosome; N_filtered_, number of bins after filtering: count of 0 per bin <20%; N_sign_, number of bins with significant correlation between the sequential (repeated) measurements (R_repeated_), with *P* < .05; N_posit_, number of bins with significant and positive correlations between the sequential measurements: *P* < .05 and R_repeated_ > 0. Correl.1 and Correl.2 are the correlations between the R_Repeated_ and R_MZ_, and R_Repeated_ and R_DZ_, respectively; Correl.3 is the correlations between R_MZ_ and R_DZ_. All the results are given as an average per chromosome.

Next, we computed intrapair correlations between all 6 501 931 bins, for MZ and DZ pairs separately, ie, R_MZ_ and R_DZ_. To explore whether the correlation between the twins depends on the bin longitudinal stability, we computed the correlation through all selected bins on each chromosome between the repeated measurements (R_repeated_) and the correlation coefficients between the twin pairs for the corresponding bins, according to their zygosity (R_MZ_ and R_DZ_), ie, R_repeated_ was contrasted with R_MZ_ (or R_DZ_).

**Figure 1.**
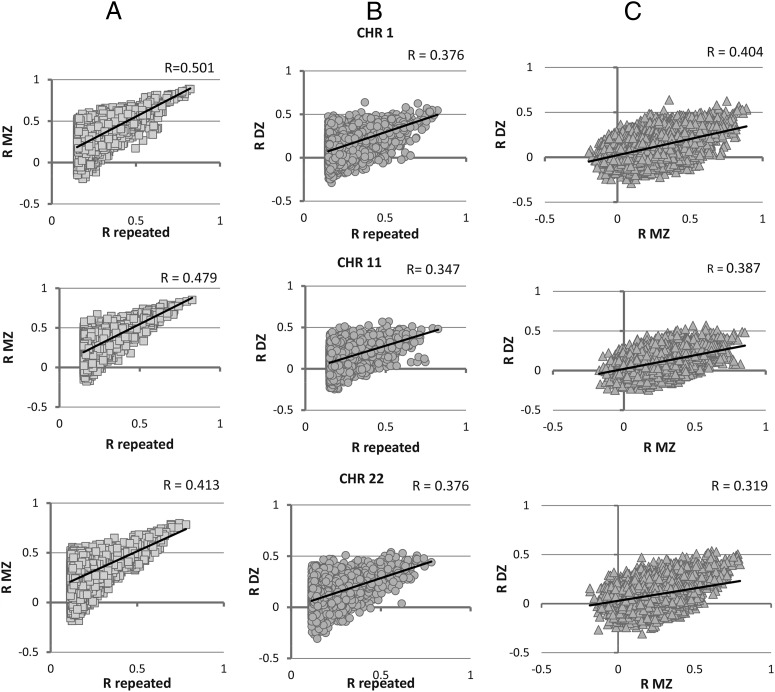
Pairwise scatterplots of correlation coefficients between the R_repeated_, R_MZ_, and R_DZ_ for the chromosomes of different size. R_repeated_, longitudinal correlations between the repeated methylation measurements per bin. R_MZ_ and R_DZ_ are intrapair correlations methylation levels per bin between the MZ and DZ twins. Columns A–C show correlation of R_repeated_ with R_MZ_ and R_DZ_ and between R_MZ_ and R_DZ_ for the selected chromosomes.

**Figure 2.**
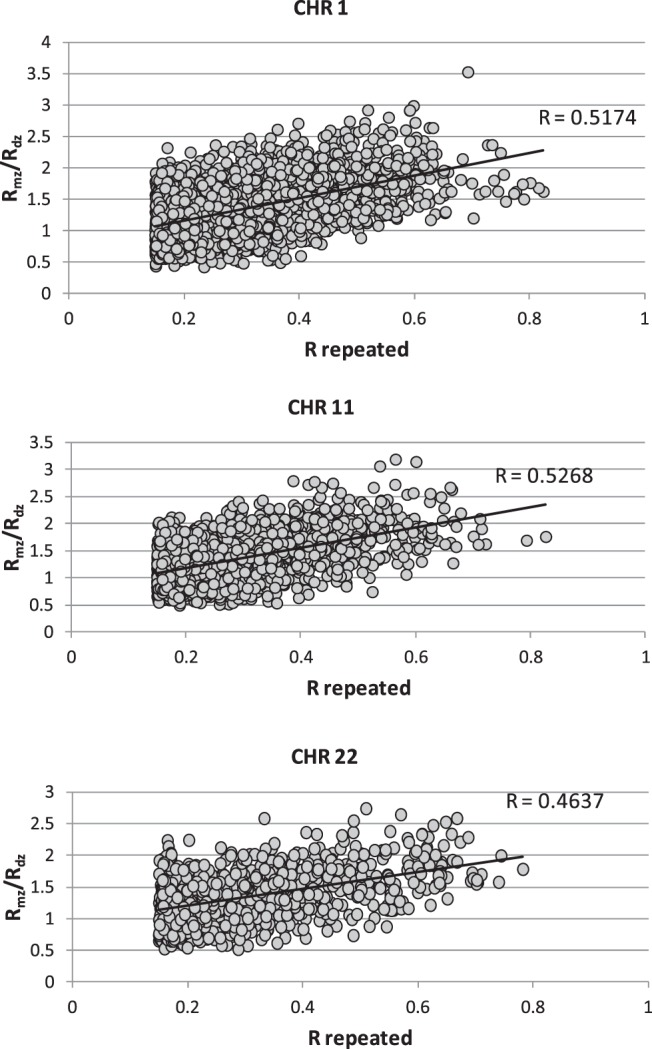
Dependence of R_MZ_ to R_DZ_ ratio on R_Repeated_. R_repeated_, longitudinal correlations between the repeated methylation measurements per bin. R_MZ_ and R_DZ_ are intrapair correlations methylation levels per bin between the MZ and DZ twins.

The correlation between the R_MZ_ and R_repeated_ was at a genome-wide average 0.51 (ranging between 0.41 and 0.58 per chromosome) and was consistently greater than the corresponding correlation between the R_DZ_ and R_repeated_, which was at a genome-wide average of 0.38 (and varied from 0.32 to 0.41 per chromosome). The corresponding results for each chromosome are provided in Table 1, and exemplifying scatterplots for chromosomes of different size are shown in Figure 1, A and B. Figure 1C demonstrates clear significant positive correlations between the R_MZ_ and R_DZ_ for the corresponding bins, with consistent tendency, R_MZ_ > R_DZ_. This relationship, expressed as R_MZ_/R_DZ_ (considering bins with positive and significant R_DZ_), shows substantial correlation (0.46–0.53, depending on chromosome) and highly significant (*P* < .0001) correlation with R_repeated_ (Figure 2). This suggests that genomic regions with evidence for genetic heritability are more likely to be longitudinally stable.

The statistical significance (*P* value) of the correlations of R_repeated_ with R_MZ_ (as well as with R_DZ_) per specific bin varied widely, depending on bin and chromosome. However, when only bins with R_repeated_ > 0 and *P* < .05 were selected, all the aforementioned correlations (ie, correlations between R_repeated_ and R_MZ_, or R_DZ_) became highly significant (*P* < 10^−8^) for all chromosomes.

### Identification of the methylation association with SMM variation in MZ twins

We tested whether the lsBINs were associated with SMM variation in the sample of 50 SMM discordant MZ twins using paired *t* test to compare methylation levels per lsBIN. Of 723 029 bin comparisons, 36 081 showed nominally significant (*P* < .05) association with SMM, which corresponds closely to 5% expected from type 1 error. These results could not be considered as entirely random, in particular for estimating the possible rate of false positive association signals. The present analysis was conducted on the small fraction of bins (6.3%), selected by QC and longitudinal stability tests. We therefore opted not to apply statistical correction technique to estimate the rate of false discovery, at this stage, as this would also increase the probability of type-2 error. Instead, of the above lsBINs, we selected only those that showed in paired *t* test for discordant MZ twins *P* < .01 and R_repeated_ > 0.4. In total, 134 autosomal bins fitted the selection criteria, and were tested in our replication sample. The distribution of the *P* values per chromosome is shown on Supplemental Figure 1, and the lsBINs from the most significant *t* tests (*P* < .001) with their genomic positions are listed in Supplemental Table 1.

### Replication of the association results in the extended population sample

At this stage, correlations of the methylation levels of the 134 aforementioned autosomal lsBINs with SMM (dependent variable) were examined in the remaining 1196 individuals. Results for 20 of them are presented on left hand side of Supplemental Table 2 as unadjusted Pearson correlations, and are arranged in the descending order for *P* values. The correction for multiple testing shows that 7 upper results (*P* values ranging between 0.0037 and 0.0003) are significant with False Discovery Rate = 0.1 (marked with bold) and are given in Table 2.

**Table 2.** Most Significant Correlations Between Appendicular Lean Mass and Methylation/Bin in Sample of 1196 Female Twins, and Multiple Regression Model for Appendicular Lean Mass as Dependent Variable and Methylation (MTL), Age and Smoking Score (pSmk) as Independent Covariates

Bin		Correlation (n = 1196)		Multiple Regression Model (n = 1100)						Nearest Gene Name and Location^a^	Gene Structure
Chr.	Start Position	R	*P*	β_MTL	P_MTL	β_Age	P_Age	β_pSmk	P_pSmk		
3	57408751	0.1051	0.0003	0.1073	0.0004	−0.0829	0.0059	0.0443	0.1404	DNAH12 57327727-530071	Intron 37 (of 58)
12	67678751	0.1022	0.0004	0.1101	0.0002	−0.0858	0.0043	0.0481	0.1100	CAND1 67663061-708472	Intron 2 (of 14)
21	15253001	−0.0918	0.0015	−0.0981	0.0011	−0.0814	0.0069	0.0392	0.1926	CYP4F29P 15215454-20685	Intergenic distance to TSS, 32.3 kb
20	50692501	−0.0917	0.0015	−0.0914	0.0023	−0.0853	0.0047	0.0378	0.2088	ZFP64 50700550-808524	Intergenic distance to TSS, 8.0 kb
3	189366751	0.0874	0.0025	0.0892	0.0030	−0.0845	0.0050	0.0401	0.1829	TP63 189349216-599284	Intron 1 (of 10)
22	24021751	−0.0852	0.0032	−0.0783	0.0095	−0.0807	0.0076	0.0356	0.2384	GUSBP11 23980675-4059610	Intron 10 (of 11)
11	76513251	0.0838	0.0037	0.0798	0.0079	−0.0850	0.0048	0.0411	0.1728	TSKU 76493357-509198	Intergenic (distance to last exon, 4 kb)

Table shows the regression coefficients, β and their corresponding *P* values (n = 1100). CALP 5 (11q13.5) belongs to the Calpain calcium-dependent cysteine proteases involved in signal transduction in a variety of cellular processes. These endopeptidases have numerous functions including, but not limited to, remodeling of cytoskeletal attachments to the plasma membrane during cell fusion and cell motility, proteolytic modification of molecules in signal transduction pathways, degradation of enzymes controlling progression through the cell cycle, regulation of gene expression. Perturbations in calpain activity have been associated in pathophysiological processes contributing to type 2 diabetes (calpain 10), muscular dystrophy (calpain 3), and other. NRIP1 (21q21.1), nuclear receptor interacting protein 1, is a nuclear protein that specifically interacts with the hormone-dependent activation domain AF2 of nuclear receptors. Also known as RIP140, this protein modulates transcriptional activity of the estrogen receptor. CPM (12q15), carboxypeptidase M, the protein encoded by this gene is a membrane-bound arginine/lysine carboxypeptidase. Its expression is associated with monocyte to macrophage differentiation. The active site residues of carboxypeptidases A and B are conserved in this protein. It is believed to play important roles in the control of peptide hormone and growth factor activity at the cell surface, and in the membrane-localized degradation of extracellular proteins. DNAH12 (3p14.3), dynein, axonemal, heavy chain 12, is a protein coding gene. Force generating protein of respiratory cilia. Produces force towards the minus ends of microtubules. Dynein has ATPase activity; the force-producing power stroke is thought to occur on release of ADP.

a Descriptions from GeneCards, http://www.genecards.org/.

We next tested whether the inclusion of the additional potential covariates, specifically age and smoking status (available for 1100 individuals) affected the aforementioned association results. The results are given on the right hand side of Table 2. Their inclusion did not change significantly the results, although age was a consistently nominally significant covariate for all the tested bins, whereas smoking did not show significant effects.

Whole blood is heterogeneous collection of cells and DNA methylation levels may reflect cellular composition. We therefore considered if the 7 top-ranked replicating SMM-association signals (Table 2) were also associated with blood cell subtypes in a subset of individuals from the replication sample, where WBC data were available. We observed no evidence for association at nominal significance (*P* = .05) between DNA methylation levels at these regions and the proportion of lymphocytes, neutrophils, basophils, and eosinophils.

We explored whether these SMM methylation results were likely caused by common genetic and/or environmental factors in SMM. We therefore conducted classical bivariate variance component analysis via estimation of contribution of pleiotropic genetic factors (genetic correlation, r_G_) and shared environmental effects (environmental correlation, r_E_) and summarized in Supplemental Table 3. It provides univariate estimates of heritability (contribution of additive genetic factors) to SMM and each of the 7 top methylation signals, and pairwise estimates of r_G_ and r_E_ between each of the bins and SMM. The heritability estimates for all bins were significant and varied between 0.271 ± 0.181 and 0.753 ± 0.091. Interesting, by Likelihood Ratio Test, genetic correlations for 5 pairs of 7 were statistically significant, with *P* values from 0.0477 to 0.0002. The remaining 2 bins on chromosome 21 (15253001-15253500) and 3 (189366751-189367250), showed nonsignificant r_G_ and r_E,_ when each of them were tested separately. However, by LRT, both correlations could not be constrained to zero (hypothesis of no correlation is rejected), with *P* = .0068 and 0.0293, suggesting that statistical power of the sample is probably insufficient to choose between r_G_ and r_E_. Other findings of interest concerned the environmental correlations (r_E_) for DNA methylation, which were all statistically nonsignificant (*P* values ranged between 0.752 and 0.068), despite the fact that initially those bins were selected in the sample of discordant MZ twins.

### Distribution of methylation signals among the CpG rich and poor genomic regions

Selected lsBINs (723 029 in total) relatively to initial total number of bins (11 524 145) were sorted as belonging to 1) CGIs, 2) CpG shores, or 3) not belonging to any of these 2 regions (CpG sea). Table 3 presents the summary of these data suggesting that there is a significant loss of lsBIN portion in CGIs (0.0017 vs 0.0032) vs clear enrichment in CpG shores (0.0525 vs 0.0285).

**Table 3.** Comparison of R_repeated_ and R_MZ_ Among the 3 Genomic Regions by CpG Content

Bins	Open Sea		Islands		Shores	
Total sample	11 159 032 [0.9683]		36 957 [0.0032]		328 156 [0.0285]	
n bins, study sample	683 823 (0.9458)^a^		1218 (0.0017)^a^		37 988 (0.0525)^a^	

	R_repeated_	R_MZ_	R_repeated_	R_MZ_	R_repeated_	R_MZ_
Average	0.1666	0.2059	0.1857	0.2478	0.1630	0.2524
Variance	0.0044	0.0175	0.0068	0.0135	0.0036	0.0141

	Comparison					
	Open Sea vs Islands		Shores vs Islands		Open Sea vs Shores	
	R_repeated_	R_MZ_	R_repeated_	R_MZ_	R_repeated_	R_MZ_
*t* test^b^	8.06	12.56	9.50	1.35#	11.32	73.88
*P* value	7.6E-16	3.5E-36	2.2E-21	1.8E-01	1.1E-29	<1.0E-300

R_repeated_, longitudinal correlations between the repeated methylation measurements per bin. R_MZ_ and R_DZ_ are intrapair correlations methylation levels per bin between the MZ and DZ twins.

a Pairwise comparison of the specific proportions of bins at each of the genomic regions by Z-test, or implementing χ^2^ test to compare the distributions of bins between the sets in all cases gave statistically highly significant results, *P* < .001 (eg, χ^2^ = 14 042, df = 2, *P* < 10^6^).

b *P* values associated with *t* tests were all (but 1, marked by #) statistically significant at *P* < .001.

However, when the distributions of R_repeated_ in these 3 regions were compared, we found that on average significantly (*P* = 2.2^−21^–7.6^−16^) higher values were observed in islands (0.1857 ± 2.4E-03) in comparison with both CGI shores (0.1630 ± 3.1E-04) and open seas (0.1666 ± 8.0E-05). With respect to R_MZ_, there were no statistically significant differences between the CGIs and shores, but both had significantly (*P* = 3.5^−36^–1.0^−300^) higher R_MZ_ in comparison with CpG seas.

### Distribution of methylation signals among functionally different genomic regions: predicted enhancers and promoters

We conducted a comparative analysis of the distribution of lsBINs mapped to predicted enhancers and promoters (Table 4, see also Supplemental Table 4). We observed statistically significantly lower levels of R_repeated_ in enhancers (average R_repeated_ = 0.1634 ± 0.0004, n = 53357) in comparison with promoters (average R_repeated_ = 0.1698 ± 0.0013, n = 8412) (t = −7.84, *P* = 4.7^−15^), as expected. Within enhancers, the percentage of lsBIN decreases (from 8.56% to 2.29%) almost monotonically (Spearman's ρ = −1.0, *P* < .0001) with the average longitudinal stability (R_repeated_) of the bin, that is, DNA methylation bins with strong evidence for longitudinal stability are less likely to fall in enhancer regions compared with DNA methylation bins with weaker evidence for longitudinal stability. The relationship with promoters, excluding the last category (where R_repeated_ > 0.7) shows the opposite trend (Spearman's ρ = 0.94, *P* < .005), but the trend is weaker if the last category is included. A similar test of the lsBIN distribution in CGIs and shores reveals a significant trend concerning the islands. In CGIs the content of lsBIN increases with their longitudinal stability, from 0.17% at R_repeated_ > 0.11 to 1.83% at R_repeated_ > 0.70 (by Spearman's ρ = 0.945, *P* = .00048), and this trend as expected is similar to that observed for promoter regions.

**Table 4.** Distribution of lsBIN Mapped Functionally Different Genomic Regions

Filtering	n Bins	Gene Function-Related Regions				CpG Content-Related Regions			
		n Within Enhancers	n Within Promoters	% in Enhancers	% in Promoters	n Within Shores	n Within Islands	% Within Shores	% Within Islands
No filtering	11 524 145	830 367	267 412	7.21%	2.32%	328 156	36 957	2.85%	0.32%
R > 0.11	723 029	61 910	16 965	8.56%	2.35%	37 988	1218	5.25%	0.17%
R > 0.2	123 984	9715	2965	7.84%	2.39%	5667	353	4.57%	0.28%
R > 0.3	35 205	2587	834	7.35%	2.37%	1325	100	3.76%	0.28%
R > 0.4	12 224	890	311	7.28%	2.54%	479	34	3.92%	0.28%
R > 0.5	4020	277	113	6.89%	2.81%	177	17	4.40%	0.42%
R > 0.6	1085	73	36	6.73%	3.32%	61	6	5.62%	0.55%
R > 0.7	218	5	4	2.29%	1.83%	9	4	4.13%	1.83%

The data provided genome wide and according to filtering conditions, R_repeated_. Spearman ρ between R_repeated_ and percentage of bins mapped to enhancers, 1.00 (*P* < .001), and percentage of bins. Mapped to CGIs, 0.964 (*P* < .0005).

## Discussion

We believe this is the first DNA methylation study to focus on SMM in a general human population. Implementing MeDIP-seq technology, we carried out a genome-wide association analysis of DNA methylation levels with SMM variation in a dataset of 1550 middle-aged females. Due to the burden of multiple testing incorporating over 10 million tests, we restricted the methylome to the methylation signals that were minimally prone to random fluctuations. We then used these selected lsBINs in an association study with SMM in 2 independent samples, to identify robust methylation signals.

After QC and analysis of longitudinal stability, our data comprised of 723 029 bins showing statistically significant correlations between repeated measurements over time. The ratio of these correlations, R_MZ_ to R_DZ_, also increased linearly with R_repeated_, suggesting that genetic factors determined this stability, as expected. This idea was further confirmed in our family-based variance component analysis of the bins most significantly associated with SMM (Supplemental Table 3), showed heritability estimates reaching 0.753 ± 0.091. Thus, our data suggest that additive genetic factors affect variation of the DNA methylation levels of only small portion of methylation sites in human genome and the magnitude of this effect determines the longitudinal stability of the bins variation.

Several studies undertaken during the past decade have estimated DNA methylation twin heritability to variation in DNA methylation at individual CpG sites across the genome (eg, Refs. 29, 30). Comparing the correlations in DNA methylation levels in blood samples between MZ and DZ twins' genome wide (26,690 CpG sites), Bell et al (24) estimated mean heritability of 0.18 (95% confidence interval, 0.168–0.185). This was confirmed by Illumina HumanMethylation450 array on peripheral blood leukocytes in a family-based sample (30). The narrow sense methylation heritability in a genome-wide analysis of 4 brain regions showed a wide range of estimates (31). The mean estimate of heritability for all available loci in the latter study was only 2.8%. However, when only loci deemed heritable were included, the mean heritability estimate was 29.9%, ranging from almost 0 to almost 1.0 (31). Remarkable was the fact that the distribution pattern of their heritability estimates was virtually identical in 15 469 loci in CGIs and in 5531 loci mapped to non-CGIs. Interestingly, only about 4% of the tested loci, in this study (31) were considered heritable. The accurate estimate of genome DNA methylation depends how exactly this proportion is calculated but is of the same order of magnitude as that observed in our study.

As mentioned the higher the heritability estimate, the more longitudinally stable is methylation site (Figure 2). Furthermore, in our study the correlations between MZ twins tended to be higher for bins located in CGIs and CGI shores in comparison with “open sea” regions (Table 3), which has not been previously explored in depth due to the limited coverage of the Illumina 450k array. In a similar vein, we observed trends with respect to the longitudinal stability of methylation bins that mapped to enhancers and promoters. The percentage of bins mapped to enhancers decreased almost monotonically with increase of R_repeated_, whereas promoters and CGIs displayed an opposite trend, as expected. These observations are in line with the observed epigenetic variability and stability at enhancers and promoters, but also suggest that longitudinally stable methylation in enhancers is a rare phenomenon that deserves further study.

It is well established that enhancers serve as distal regulators of gene expression. Enhancer methylation is drastically altered in cancers and is closely related to altered expression profiles of cancer genes (eg, Refs. 32–34), suggesting that enhancer hypo-/hypermethylation could be critical in transition from normal to aberrant status of the cells. Thus, it looks that the low content of longitudinally stable methylation sites mapped to enhancers play a protective role in transcription regulation of the structural genes.

Our 2-stage association analysis suggests that the methylation status of 5–7 regions is significantly associated with variation of SMM. The peak 7 regions mapped predominantly within gene bodies (4 of 7) and near to the gene transcription start site (TSS) (within 10 kb), with the exception of the intergenic signal nearest to the *CAND1* gene (32 kb to TSS). Of the 7 reported genes, the top-ranked SMM signal was located within the *DNAH12* gene, which is expressed in vastus lateralis muscle tissue after 20 weeks of endurance exercise training (35). In mice, Zfp64 has been demonstrated to participate in regulation of mesenchymal cell differentiation by suppression of myogenic, and promotion of osteoblastic, cell fate through Notch signaling (36). Tp63 has been shown to be a marker for skeletal muscle differentiation (37) and is also used for defining subsets of muscle-invasive bladder cancers (38). Finally, *GUSBP11* has been listed in the C480 FANTOM5 coexpression cluster with numerous muscle-related top-ranked ontology terms such as skeletal muscle, contractile cell, myoblasts, striated muscle tissue, myotome, etc (39). Hence, although the current study was conducted using whole blood, 4 of the 7 reported genes had previously been recognized in muscle-related studies, suggesting that the methylome of whole blood is informative of SMM variation.

Currently, as far as we aware, no SNPs association at genome-wide association study (GWAS) significant level (*P* < 5 × 10^−8^) have been reported. Nevertheless, of the several recent studies, one by Guo et al (40) provides evidence of significant association of 3 SNPs (*rs2507838*, *rs7116722*, and *rs11826261*) in/near glycine-N-acyltransferase (*GLYAT*) gene in Chinese and in Caucasian populations. However, this gene is mapped, too far away (∼2000 kb) to consider as a reliable candidate. Thus, clearly additional GWAS and EWAS (epigenome-wide association study) are needed to understand sarcopenia.

There are certain limitations in the present study. The main potential bias concerns the usage of blood. Whole blood includes a heterogeneous group of cells whose composition can change substantially in response to a variety of intrinsic and environmental factors, eg, infection. We assessed the association between DNA methylation levels at the 7 SMM-associated and replicated DNA methylation loci with blood cell counts available for the proportion of lymphocytes, neutrophils, basophils, and eosinophils in a subset. We found no evidence that variability in WBC subtypes has a major effect on our top-ranked muscle mass related signals. The fact that we were also able to replicate these association results in a large independent sample was reassuring, and suggests that whole blood may be informative of SMM variation. MeDIP-seq provides a cost-effective approach to exploring broad methylation patterns over the genome, but the resolution of the signal is not at single base pair level CpG sites. This is an additional major limitation in interpreting the methylation signals, and requires a follow up of the identified signals at greater resolution in future studies. Additional limitations include using a female population only, because males were not examined. However, using only females, could have possible advantages This approach is not prone to potential sex differences in endocrine milieu, especially sex steroids, lifestyle factors, etc, and their interactions with genotype and epigenotype, which tend to be the rule rather than the exception (41, 42). Physical activity level, other lifestyle factors, medications and other environmental factors were not taken into account in this study, and may have affected the magnitude of the estimated effects. Finally, there are also some limitations related to the power of the analysis. To achieve EWAS significance accounting for multiple testing at millions of DNA methylation CpG sites would require very large sample sizes or very large effects, which have not been observed to date. To this end our association analysis was based only the longitudinally stable and most robust fraction of the methylome (<6.5% of the initial methylome).

## Conclusions

This large EWAS study confirmed a major involvement of the genetic factors (heritability) to variation of SMM in a normal human population, and suggested that methylation levels of some genomic regions could underlie this variation. Four of our 7 muscle mass associated methylation signals were in or near genes previously linked to muscle, and in particular the top-ranked *DNAH12* gene. Our approach based on a selection of the lsBINs reduces substantially the multiple testing problem and proved to be effective in identification of the methylation sites potentially affecting SMM variation.
